# The role of social support and psychological capital in college students' physical exercise behavior: a mediation analysis

**DOI:** 10.3389/fpsyg.2025.1601079

**Published:** 2025-07-30

**Authors:** You Xiong, Juncheng Si, Zhan Huang, Junjie Ouyang, Shuai Ma, Yao Huang, Xiaoling Huang

**Affiliations:** College of Physical Education, Southwest University, Chongqing, China

**Keywords:** physical exercise behavior, college students, social support, psychological capital, mediation analysis

## Abstract

**Background:**

The physical exercise behavior of college students is closely related to their physical and mental health. Research has shown that social support and psychological capital are related to college students' physical exercise behavior, but their correlation needs further exploration.

**Objectives:**

This study seeks to investigate the relationship between social support, psychological capital and university students' physical exercise behavior, with a further in-depth analysis of the mediating roles played by psychological capital within this dynamic.

**Methods:**

Using cluster sampling, we surveyed 359 college students in Southwest University, employing the Sports Activity Level Scale (SALS), the Positive Psychological Capital Questionnaire (PPCQ), the Social Support Scale (SSS). Data were analyzed using SPSS 26.0 and STATA 17.0, and common method bias was tested using Harman's single-factor test. The significance of the mediation effects was tested using the Kohler, Karlson, and Breen (KHB).

**Results:**

(1) Peer support (*P* < 0.01), teacher support (*P* < 0.01), family support (*P* < 0.05), and psychological capital (*P* < 0.05) are significantly correlated with college students‘ physical exercise behavior. (2) Social support positively correlates with the positive psychological capital of college students (*P* < 0.01). The indirect effects of peer, teacher, and family support on college students' physical exercise behavior through psychological capital account for 87.34%, 36.95%, and 30.69% of the total effect. (3) According to the regression coefficient, social support is ranked as peer support (0.11), teacher support (0.10), and family support (0.09).

**Conclusion:**

Social support shows a positive association with college students' physical exercise behavior and also demonstrates a significant correlation with it through psychological capital. Teacher support in the school living environment is equally important, and future research should focus on developing the role of family support.

## Introduction

In recent years, due to the lack of physical exercise and participation enthusiasm of college students, the physical health level of college students in China has been low, and their physical quality has shown a downward trend (Duncan et al., 2007). College students are the builders of the future society. Stimulating college students to adhere to physical exercise will help improve the quality of talent training in China's higher education and promote the implementation effect of the “healthy China 2030” strategy (Tang and Wang, 2024). Colleges and universities pay more and more attention to students‘ physical quality, and often carry out a series of after-school training, competitions and other activities. College students' participation in sports is not only the requirement of the current society for the comprehensive development of college students, but also an important way to promote college students‘ Lifelong Sports consciousness (LaCount et al., 2022). Therefore, paying attention to college students' physical exercise behavior is also an important social problem.

With the proposal of the concept of healthy China and the development of behavioral psychology, many scholars have studied the characteristics of College Students' physical exercise behavior, the internal and external factors affecting college students' physical exercise behavior, and the mechanism of physical exercise behavior. Research has confirmed the applicability of psychological capital theory and social support theory in the field of physical exercise. Social support theory holds that all kinds of support people get during physical exercise will help to enhance people's enthusiasm to participate in physical exercise, so as to encourage people to participate in physical exercise more (Mathew et al., 2022). It refers to the material or spiritual support that can be obtained through social connections. For example, material assistance or direct services, emotional support (Warner et al., 2011). At present, scholars have divided social support into different categories according to the different environments of the research objects. Ren et al. studied the impact of physical exercise on individual social anxiety in children. They divided social support into family support, friend support and other support (Ren and Li, 2020). Belanger et al. studied the impact of different sources and types of social support on College Students' sports behavior, and the author considered peer support and family support (Belanger and Patrick). Zhang et al. studied the impact of social support on College Students' willingness to participate in physical exercise, mainly evaluating the role of friends, family and campus sports cultural atmosphere in sports activities (Zhang et al., 2022). However, the above research ignored the importance of teacher support. Some scholars have found that the lack of teacher support is one of the important factors affecting students' physical exercise (Yan et al., 2025). The social support that students get from PE teachers can encourage students to look for similar activities outside school in their spare time. The more teachers support students to participate in physical exercise, the higher the students' autonomous motivation inside and outside the classroom (Abula et al., 2020). Therefore, in this study, teacher support was added to the previous two categories of family and peer support, and social support was examined from three perspectives: teacher support, peer support, and family support. Regarding the definition of the concept, teacher support refers to the shaping of students' health concept, the guidance of physical exercise behavior, and the training of sports skills. Peer support refers to the supportive behavior of relevant partners for individual physical exercise behaviors. Family support refers to parents' exercise behavior, parents' financial support, and family fitness equipment (Dan and Yao, 2015).

The concept of “positive psychology” was first proposed more than 20 years ago (Seligman and Csikszentmihalyi, 2000). At the individual level, positive psychology refers to positive individual characteristics, including love and mission, courage, interpersonal skills, perseverance, tolerance, originality, future consciousness, spirituality, talent and wisdom. The concept of “psychological capital” is based on the concept of positive psychology and is used to express the personal motivation tendency generated through the positive psychological structure, including four dimensions of self-efficacy, hope, optimism and resilience (Luthans and Youssef, 2004). Psychological capital is an important factor for individuals to obtain competitive advantage, implement positive behavior and achieve good performance (Luthans et al., 2007). As a positive psychological trait, psychological capital is based on positive emotions and emotional experience. Psychological capital is of great significance in the initial process of people's participation in physical exercise. The higher the content of self-efficacy in psychological capital, the better the performance in the initial stage of physical exercise. This is because psychological capital can help participants overcome some obstacles of physical exercise, such as overcoming learning pressure and insisting on physical exercise. A positive psychological state is positively associated with increased confidence and enthusiasm for college students' active engagement in physical exercise (Motl et al., 2007).

There have been a lot of research results on College Students‘ group physical exercise behavior, but there are few studies to explore the influencing factors of College Students' group physical exercise based on the combination of social support theory and psychological capital theory, which largely ignores the important significance of teacher support. Therefore, this study attempts to start from the social support theory and psychological capital theory, and deeply studies the path relationship between the two in college students‘ physical exercise behavior, which provides an important theoretical basis for promoting college students' autonomy and persistence in physical exercise.

## Research hypothesis

### The relationship between social support and physical exercise behavior

Social support is a key variable affecting young people's physical exercise activities (Courneya et al., 2000). Adequate social support is an effective way to cultivate college students to participate in physical exercise (Alshehri et al., 2021). Dong et al. found that social support can buffer the negative impact of physical exercise discomfort on the body and mind, and enhance the sense of pleasure and satisfaction brought by exercise (Dong et al., 2018). Family support and significant others support as important factors for exercise adherence behavior (Courneya and Mcauley, 1995). College students who perceive social support can gain intrinsic motivation to actively participate in physical exercise. Based on this, this study proposes the following hypotheses:

H1: Teacher support has a significant positive effect on college students' physical exercise behavior.
H2: Peer support has a significant positive effect on college students' physical exercise behavior.
H3: Family support has a significant positive effect on college students' physical exercise behavior.

### The relationship between social support and psychological capital

Social support and psychological capital are not independent of each other. The theory of positive psychological capital proposes that supportive environments have a positive impact on psychological capital (Luthans et al., 2008). Psychological capital can regulate the impact of social support on physical exercise, and people with higher hope, self-efficacy, and respect benefit more from support, making them more likely to convert support into physical exercise behavior (Kang et al., 2019). Research has shown that a satisfactory social environment and interpersonal support are beneficial for increasing individuals' confidence in participating in sports activities (Ren et al., 2020). Therefore, the following hypotheses are proposed:

H4: Teacher support has a significant positive effect on psychological capital.
H5: Peer support has a significant positive effect on psychological capital.
H6: Family support has a significant positive effect on psychological capital.

### The relationship between psychological capital and physical exercise behavior

Psychological capital has a certain correlation with behavioral variables. By increasing the content of individual psychological capital, it can effectively stimulate people's subjective initiative and provide a continuous source of motivation for the sustainability of individual physical exercise behavior (Teixeira et al., 2012). The higher the psychological capital content of college students, the more positive their attitude toward physical exercise is. The easier it is for them to persist in physical exercise, which helps to shape a healthy personality (Yang et al., 2013). Li et al. also believes that the higher the level of psychological capital among college students, the higher their attitude toward sports, which in turn promotes good physical exercise behavior among individuals (Li, 2019). Therefore, the following hypotheses are proposed:

H7: Psychological capital has a significant positive impact on physical exercise behavior.

**Figure 1:**
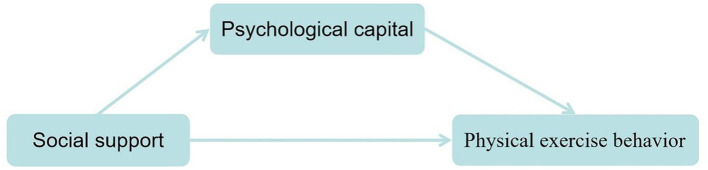
Conceptual model.

In summary, in order to explore the relationship between social support, psychological capital, and physical exercise behavior, this study aims to construct a chain mediation model (Figure 1).

## Materials and methods

### Procedure and participants

A questionnaire survey was conducted among college students in Chongqing using both on-site and online methods. The survey was carried out in two stages. In the first stage, 145 students from two classes at Southwest University were selected for the survey. After removing 10 invalid questionnaires, 135 valid questionnaires were obtained and used for reliability and validity testing. Upon successful completion of the testing, the second stage of the formal investigation commenced. In the second stage, a random sampling method was employed, with a total of 385 questionnaires distributed and 359 valid questionnaires collected. The responses to the questions were measured using a 5-point Likert scale, where 1–5 points corresponded to “strongly agree,” “agree,” “neutral,” “disagree,” and “strongly disagree,” respectively. When analyzing the physical exercise behavior of college students, the duration, intensity, and frequency of independent physical exercise were considered, and scores ranging from 1 to 5 were assigned.

### Measures and instruments

All the scales employed have corresponding Chinese versions, and the following offers a detailed elaboration.

#### Physical exercise rating scale

In the process of measuring the physical exercise behavior of college students, the “Sports Activity Level Scale” designed by Liang (1994) was adopted as the primary indicator. This scale evaluates individual physical exercise behavior across three dimensions: frequency, intensity, and duration of physical exercise (Liang, 1994). Based on previous research, this study chose the frequency of physical exercise as the measurement indicator (Slade and Keating, 2012). The frequency of physical exercise includes five items (such as no exercise, several times a year, several times a month, several times a week, and daily exercise), where 1 represents no exercise, 2 represents several times a year, 3 represents several times a month, 4 represents several times a week, and 5 represents daily exercise. The comprehensive assessment provided by this scale ensures a holistic understanding of students' exercise habits.

#### Psychological capital scale

Psychological capital was measured using the Positive Psychological Capital Questionnaire, compiled by Zhang et al. (2010) from the Department of Social Psychology at Nankai University. This questionnaire comprises four dimensions: self-efficacy, resilience, hope, and optimism, with a total of 26 items (Zhang et al., 2010). A 5-point Likert scale was employed, ranging from 1 (completely disagree) to 5 (completely agree). When measuring, use principal component analysis to calculate the total score of the scale. Higher total scores indicate better psychological states in students' academic and life contexts. In this study, the scale demonstrated high reliability, with a Cronbach's α of 0.93.

#### Social support scale

Social support was assessed using the Social Support Scale developed by Chen et al. (2016). Originally divided into three dimensions—family, friends, and others—this scale was revised and localized for the current study. The revised version includes three dimensions: family support, peer support, and teacher support, with a total of 12 items (Chen et al., 2016). Responses were recorded on a 5-point Likert scale, where 1 represented “completely disagree” and 5 represented “completely agree.” When measuring, use principal component analysis to calculate the total score of social support for families, peers, and teachers. The higher the total score, the higher the individual's support rate from that dimension. The scale exhibited excellent reliability in this study, with a Cronbach's alpha of 0.94.

### Data analysis

Data analysis was conducted using IBM SPSS 26.0 and STATA 17.0. The following steps were taken to ensure the robustness and validity of the results:

#### Common method bias test

To inspect potential common method bias, Harman's single-factor test was employed. All items were subjected to an unrotated exploratory factor analysis after statistical dimensionality reduction. The results revealed 10 factors with eigenvalues >1.0, with the largest factor explaining 39.27% of the variance—below the 40% threshold (Tang and Wen, 2020). Thus, no significant common method bias was detected in this study.

#### Descriptive statistics and reliability analysis

IBM SPSS 26.0 was used for initial data analysis. Descriptive statistics were calculated for key variables, including social support, psychological capital, and physical exercise behavior. Internal consistency reliability was assessed using Cronbach's alpha, while convergent validity was evaluated through Kaiser-Meyer-Olkin (KMO) and Bartlett's sphericity tests.

#### Principal component analysis and multiple regression

STATA 17.0 was utilized for advanced statistical analysis. Principal component analysis (PCA) was conducted to construct relevant indicators. Subsequently, multiple regression analysis was performed to explore the interrelationships between social support, psychological capital, and physical exercise behavior.

#### Mediation effect analysis

The mediation effect of psychological capital was tested using the Kohler, Karlson, and Breen (KHB) method. This approach estimates the coefficients of total effects, direct effects, and indirect effects to calculate the proportion of mediating effects. The KHB method is applicable to binary dependent variables. In the mediation effect analysis section, we transform physical exercise behavior into binary classification for analysis, where 0 represents never exercise, 1 represents several times a year, several times a month, several times a week, and daily exercise. The analysis aimed to evaluate the mediating role of psychological capital in the relationship between social support and college students' physical exercise behavior.

## Result

### General characteristics of participants

Among the 359 participants, those aged 18–22 accounted for 71.87% of the total, with a higher proportion of males (57.10%) than females. In addition, students majoring in psychology, education, agriculture, pharmacy, and other fields account for 65.18%, while students majoring in sports account for 34.82%.

### Results of confirmatory factor analysis

Confirmatory factor analysis (CFA) was used to verify the construct validity. When AVE > 0.5 and CR > 0.7, it indicated convergent validity and composite reliability (Sum et al., 2018). Independent confirmatory factor analysis was conducted on peer support, teacher support, family support, psychological capital and physical exercise behavior respectively. After eliminating the indexes with factor load < 0.5, the results showed that peer support (AVE = 0.72, CR = 0.89), teacher support (AVE = 0.75, CR = 0.93), parent support (AVE = 0.73, CR = 0.92), psychological capital (AVE = 0.50, CR = 0.98), physical exercise behavior (AVE = 0.76, CR = 0.92) show that the model converges effectively. In addition, we examined the degree of fit between the measurement model and the actual collected data, and the results showed that χ2/df = 3.638, RMESA = 0.052, GFI = 0.913, AGFI = 0.901, NFI = 0.923, CFI = 0.942, IFI = 0.943. All the fitting indexes are higher than the standard of 0.9, indicating a good degree of fitting.

### Descriptive statistical

#### Social support and psychological capital

Table 1 reveals that, in the comparison of social support among college students of different genders, both male and female students scored highest in peer support, with scores of 76.994 and 77.296, respectively. Conversely, they scored lowest in parental support, with scores of 75.810 for males and 70.084 for females. Regarding gender differences, no significant disparities were observed in peer support and teacher support; however, a statistically significant difference was found in family support (*P* < 0.05). In the comparison of psychological capital across genders, male college students exhibited a higher psychological capital score (mean = 70.447) compared to female students (mean = 68.120).

**Table 1:** Social support and psychological capital of college students between different genders.

**Dimensions**	**Gender**	**Number**	**M ±SD**	**T**	***P***
Peer support	Female	154	77.296 ± 1.855	0.127	0.898
	Male	205	76.994 ± 1.513		
Teacher support	Female	154	73.433 ± 2.062	−0.986	0.324
	Male	205	76.002 ± 1.643		
Family support	Female	154	70.840 ± 1.902	−2.009	0.045^*^
	Male	205	75.810 ± 1.597		
Psychological capital	Female	154	68.120 ± 1.708	−1.064	0.288
	Male	205	70.447 ± 1.394		

^*^ indicating significant differences.

#### Physical exercise

As shown in Table 2, the proportion of male college students participating in physical exercise weekly is 19.5%, compared to 14.8% for female students. Regarding exercise intensity, male students predominantly engage in high-intensity training, accounting for 25.9%, while female students primarily opt for moderate-intensity training, representing 20.1%. In terms of exercise duration, the vast majority of male students (24.7%) exercise for more than 60 min per session, whereas most female students (17.6%) exercise for 30–59 min per session.

**Table 2:** Physical exercise of college students between different genders.

**Indicators**		**Gender**	**Number**	**Account for**	**Total**
Frequency	Never exercise	Female	4	1.1%	7 (1.9%)
		Male	3	0.8%	
	Several time a year	Female	17	4.7%	36 (10.0%)
		Male	19	5.3%	
	How many times a month	Female	53	14.8%	117 (32.6%)
		Male	64	17.8%	
	Several times a week	Female	52	14.5%	122 (34.0%)
		Male	70	19.5%	
	Exercise every day	Female	28	7.8%	77 (21.4%)
		Male	49	13.6%	
Intensity	Low intensity	Female	26	7.4%	63 (17.9%)
		Male	37	10.5%	
	Medial	Female	71	20.1%	145 (41.1%)
		Male	74	21.0%	
	High intensity	Female	53	15.1%	144 (41.0%)
		Male	91	25.9%	
Duration	0–30 min	Female	43	12.2%	74 (21.0%)
		Male	31	8.8%	
	30–59 min	Female	62	17.6%	146 (41.5%)
		Male	84	23.9%	
	More than 60 min	Female	45	12.8%	132 (37.5%)
		Male	87	24.7%	

### Empirical results analysis

#### Regression analysis of social support on college students' physical exercise behavior

Table 3 presents seven models examining the association between social support and college students‘ physical exercise behavior. Model 1 primarily reflects the correlation of control variables with sports participation behavior. Models 2, 4, and 6 serve as baseline models, incorporating only the independent variables (peer support, teacher support, family support) and the dependent variable (college students' physical exercise behavior). Models 3, 5, and 7 expand upon the baseline models by including all control variables. The regression results indicate that:

- A one-unit increase in peer support leads to a 10.6% increase in the frequency of college students' physical exercise participation (*P* < 0.01).
- A one-unit increase in teacher support results in a 10.2% increase in the frequency of physical exercise participation (*P* < 0.01).
- A one-unit increase in family support corresponds to an 8.7% increase in the frequency of physical exercise participation (*P* < 0.05).

**Table 3:** The impact of social support on college students' physical exercise behavior.

**Dependent variable: college students' physical exercise behavior**							
**Variables**	**Model 1**	**Model 2**	**Model 3**	**Model 4**	**Model 5**	**Model 6**	**Model 7**
Peer support		0.108^**^	0.106^**^				
		(0.047)	(0.046)				
Teacher support				0.101^**^	0.102^**^		
				(0.045)	(0.045)		
Family support						0.080^*^	0.087^*^
						(0.046)	(0.047)
Gender	0.269		0.237		0.258		0.291
	(0.196)		(0.197)		(0.197)		(0.197)
Major	0.046		0.000		0.028		0.029
	(0.208)		(0.209)		(0.208)		(0.208)
Grade	−0.008		0.022		0.004		−0.045
	(0.220)		(0.221)		(0.220)		(0.221)
*N*	359	359	359	359	359	359	359
pseudo *R*^2^	0.003	0.006	0.008	0.005	0.008	0.003	0.007

^*^, ^**^ respectively indicate significance at the 0.05 and 0.01 levels.

Specifically, peer, teacher, and family support are positively correlated with college students' engagement in physical exercise, with peer and teacher support showing stronger associations. Therefore, hypotheses H1, H2, and H3 are supported.

#### Regression analysis of social support on psychological capital

Table 4 presents seven models examining the impact of social support on psychological capital. Model 1 primarily reflects the association between control variables and psychological capital. while Models 2, 4, and 6 serve as baseline models, incorporating only the independent variables (peer support, teacher support, family support) and the mediating variable (psychological capital). Models 3, 5, and 7 expand upon the baseline models by including all control variables.

**Table 4:** The impact of social support on psychological capital.

**Dependent variable: psychological capital**							
**Variables**	**Model 1**	**Model 2**	**Model 3**	**Model 4**	**Model 5**	**Model 6**	**Model 7**
Peer support		1.066^**^	1.067^**^				
		(0.043)	(0.043)				
Teacher support				1.038^**^	1.040^**^		
				(0.043)	(0.043)		
Family support						0.984^**^	1.001^**^
						(0.049)	(0.049)
Gender	0.310		0.369^*^		0.080		−0.087
	(0.314)		(0.191)		(0.193)		(0.212)
Major	0.181		−0.050		0.010		−0.165
	(0.335)		(0.204)		(0.206)		(0.226)
Grade	0.377		0.011		0.565^**^		0.731^***^
	(0.357)		(0.218)		(0.220)		(0.242)
*N*	359	359	359	359	359	359	359
*R* ^2^	0.011	0.632	0.636	0.618	0.625	0.534	0.551

^*^, ^**^ respectively indicate significance at the 0.05 and 0.01 levels.

The regression results demonstrate that peer, teacher, and family support are significantly positively correlated with college students' positive psychological capital (*P* < 0.01). Thus, hypotheses H4, H5, and H6 are supported. Moreover, the magnitude of the regression coefficients indicates that peer support (β = 1.07) shows the strongest association with psychological capital, followed by teacher support (β = 1.04) and family support (β = 1.0).

#### Regression analysis of psychological capital on college students' physical exercise behavior

Table 5 presents seven models examining the impact of psychological capital on college students‘ exercise behavior. Model 1 primarily reflects the association between control variables and physical exercise behavior, while Model 2 serves as the baseline model, incorporating only the mediating variable (psychological capital) and the dependent variable (college students' physical exercise behavior). Model 3 expands upon the baseline model by including all control variables.

**Table 5:** The association between psychological capital and college students' physical exercise behavior.

**Dependent variable: college students' physical exercise** **behavior**			
**Variables**	**Model 1**	**Model 2**	**Model 3**
Psychological capital		0.074^*^	0.075^*^
		(0.034)	(0.035)
Gender	0.269		0.257
	(0.196)		(0.197)
Major	0.046		0.029
	(0.208)		(0.208)
Grade	−0.008		−0.042
	(0.220)		(0.221)
*N*	359	359	359
pseudo *R*^2^	0.003	0.005	0.008

^*^ indicate significance at the 0.05 levels.

The regression results demonstrate that a one-unit increase in psychological capital leads to a 7.5% increase in the opportunity for college students to participate in physical exercise (*P* < 0.05). This indicates that psychological capital has a significant positive effect on promoting physical exercise behavior among college students. Therefore, hypothesis H7 is supported.

### Mediation effect results

Table 6 examines the mediating role of psychological capital. The results show that peer support, teacher support, and family support are significantly positively correlated with psychological capital (*P* < 0.01). The indirect effects of social support on college students' physical exercise behavior, mediated by psychological capital, account for 87.34%, 36.95%, and 30.69% of the total effects, respectively. In other words:

- 87.34% of the impact of peer support on physical exercise behavior is achieved through enhancing psychological capital.
- 36.95% of the impact of teacher support on physical exercise behavior is achieved through enhancing psychological capital.
- 30.69% of the impact of family support on physical exercise behavior is achieved through enhancing psychological capital.

**Table 6:** Mediating effect of psychological capital.

**Route**	**Direct effect**	**Indirect effect**	**Total effect**	**Proportion of mediating effect (%)**
Peer support → Psychological capital → College students' physical exercise behavior	0.010^**^	0.069^**^	0.079^**^	87.34
Teacher support → Psychological capital → College students' physical exercise behavior	0.058^**^	0.034^**^	0.092^**^	36.95
Family support → Psychological capital → College students' physical exercise behavior	0.070^**^	0.031^**^	0.101^**^	30.69

^**^ indicate significance at the 0.01 levels.

These findings confirm that psychological capital plays a significant mediating role in the relationship between social support and college students' physical exercise behavior.

## Discussion

### The association between peer support and college students' physical exercise behavior

The results indicate that peer support shows the strongest positive correlation with college students' physical exercise behavior. Peer relationships play a crucial role in motivating college students to engage in physical exercise (Bull et al., 2020). which can be attributed to the following reasons: First, the similarity in health behaviors among peers and the exemplary role of peers effectively encourage individual participation in sports activities (Ji et al., 2022). Specifically, when college students join various activities or sports clubs, the sports atmosphere created by their peers often inspires them to participate in physical exercise collectively. Additionally, peer support is positively associated with college students' happiness and satisfaction during physical exercise, fostering positive emotional experiences, boosting confidence, and facilitating the acquisition of new sports skills (Chen et al., 2017). Haidar et al., focusing on college students as their research subjects, explored the relationship between peer support and physical exercise. Their findings indicate that every unit of peer support increases the likelihood of college students participating in physical activities by at least 1.15 times (Haidar et al., 2019). The findings of this paper align with the conclusions of the aforementioned scholars, confirming that peer support has a significant positive impact on college students' physical exercise behavior.

### The association between teacher support on college students' physical exercise behavior

The association between teacher support on college students' physical exercise behavior is second only to peer support. For students, school is the most significant environment in their daily lives and the primary setting where they are likely to engage in physical exercise. Consequently, sports activities exhibit strong correlations with multiple facets of school life, including the promotion of students' values and the shaping of their ideological consciousness. Teachers, as direct transmitters of knowledge and values, play a pivotal role in students' development. Perceived teacher support is a key variable positively correlated with personal psychological factors—including goal orientation and achievement motivation (Ghorbani et al., 2020). I Moreover, teacher support helps students better understand motor skills and reduces psychological barriers to participation. For instance, teachers design exercises and competitive activities tailored to students' ability levels, enabling them to experience moderate challenges and a sense of achievement, thereby enhancing their interest in sports (Liu et al., 2025). Chatzisarantis et al. conducted a large-scale intervention study, confirming the positive impact of teacher support on students' participation in extracurricular sports activities. Compared to students under neutral teacher support conditions, those receiving active teacher support demonstrated a stronger willingness to exercise during leisure time and participated in sports activities more frequently (Chatzisarantis and Hagger, 2009). Therefore, teacher support not only provides students with a sense of security but also significantly promotes their engagement in physical exercise.

### The association between family support on college students' physical exercise behavior

Compared with teacher support and peer support, family support has a relatively weaker impact on college students' physical exercise behavior. While the family is a primary environment for children's lives alongside school, it remains an important source of support for college students' physical activity behavior (Belanger and Patrick, 2018). Research indicates that family support positively promotes adolescents‘ participation in sports activities, adherence to physical exercise routines, and acquisition of sports skills (Jiang and Xiao, 2024). Dong et al. found that parental support enhances adolescents' control beliefs, not only encouraging their active participation in exercise but also influencing their level of engagement and strengthening their persistence in exercise (Dong and Mao, 2018). However, during college, factors like physical distance and emotional independence weaken the association between family support and students‘ physical exercise behavior. This shift in dynamics explains why family support plays a less prominent role in shaping college students' exercise behavior compared to peer and teacher support.

### The mediating effect of psychological capital

Further research has revealed that social support can indirectly predict college students' physical exercise behavior through the mediating effect of psychological capital. The dual engines driving college students' physical exercise behavior are internal psychological appeals and external perceptual support. The interaction between these internal and external factors effectively enhances college students' health awareness and behavior (Dong et al., 2018). The more support students receive from significant figures during fitness activities, the stronger their self-esteem protection becomes. This confidence helps them eliminate negative emotions during sports activities, thereby increasing their psychological capital and fostering self-awareness and autonomy in exercise (Zhou et al., 2014). In this study, teacher support, peer support, and family support were found to promote physical exercise behavior among college students by enhancing their psychological capital, including self-efficacy, resilience, hope, and optimism. Specifically, teacher support is positively associated with students' self-efficacy, which in turn correlates with greater enthusiasm for sports participation (Dutrisac et al., 2023). Peer support strengthens students' optimism through emotional, informational, and instrumental support, which further promotes individual physical exercise behavior (Luo et al., 2022). Family support, particularly through a positive sports atmosphere, significantly increases students' confidence in physical exercise and encourages their participation and persistence in such activities (Jiang and Xiao, 2024). These findings confirm that psychological capital plays a mediating role in promoting college students' physical exercise behavior. The mediation model constructed in this study is feasible and provides valuable insights into the internal mechanisms through which social support improves college students' physical exercise behavior. Additionally, it offers practical guidance for initiatives aimed at enhancing college students' engagement in physical exercise.

### Limitations

The results of this study are limited to the mediating role of psychological capital in the relationship between social support and physical exercise behavior among college students. Other potential mediating variables, such as family environment, self-control, and health concepts, remain unexplored and warrant further investigation in subsequent research. Additionally, the study focuses exclusively on college students over 19 years old, resulting in a relatively homogeneous sample. Caution is necessary when extending variables such as social support and physical exercise behavior to other age groups. Future research should aim to expand the sample range to include diverse populations. Furthermore, this study relies on self-reported data from college students, which may introduce method bias and affect the validity of the findings. To address this limitation, future research could consider incorporating perspectives from teachers, peers, and parents as additional research subjects. This multi-source approach would provide a more comprehensive understanding of the relationships between the variables.

## Conclusion

This study takes college students in Chongqing as the research object, constructs a theoretical framework of social support, psychological capital, and college students' physical exercise behavior. Research has found that: (1) Social support and enhanced psychological capital are positively correlated with college students' physical exercise behavior. (2) Psychological capital serves as a significant mediator in the indirect association between social support and college students‘ physical exercise behavior. (3) As for the effectiveness of social support, peer support has the greatest impact on college students' physical exercise behavior, followed by teachers, and finally family members.

## Data Availability

The raw data supporting the conclusions of this article will be made available by the authors, without undue reservation.
